# Mild Traumatic Brain Injury in Adolescent Mice Alters Skull Bone Properties to Influence a Subsequent Brain Impact at Adulthood: A Pilot Study

**DOI:** 10.3389/fneur.2018.00372

**Published:** 2018-05-25

**Authors:** Thomas J. McColl, Rhys D. Brady, Sandy R. Shultz, Lauren Lovick, Kyria M. Webster, Mujun Sun, Stuart J. McDonald, Terence J. O’Brien, Bridgette D. Semple

**Affiliations:** ^1^Department of Medicine, Royal Melbourne Hospital, University of Melbourne, Parkville, VIC, Australia; ^2^Department of Physiology, Anatomy and Microbiology, La Trobe University, Bundoora, VIC, Australia; ^3^Department of Neuroscience, Central Clinical School, Monash University, Melbourne, VIC, Australia

**Keywords:** mild traumatic brain injury, mouse model, inflammation, behavior, bone

## Abstract

Mild traumatic brain injuries (mTBI) are common during adolescence, and limited clinical evidence suggests that a younger age at first exposure to a mTBI may lead to worse long-term outcomes. In this study, we hypothesized that a mTBI during adolescence would predispose toward poorer neurobehavioral and neuropathological outcomes after a subsequent injury at adulthood. Mice received a mild weight drop injury (mTBI) at adolescence (postnatal day 35; P35) and/or at adulthood (P70). Mice were randomized to 6 groups: *‘sham’* (sham-surgery at P35 only); *‘P35’* (mTBI at P35 only); *‘P35* + *sham’* (mTBI at P35 + sham at P70); *‘sham* + *P70’* (sham at P35 + mTBI at P70); *‘sham* + *sham’* (sham at both P35 and P70); or *‘P35* + *P70’* (mTBI at both P35 and P70). Acute apnea and an extended righting reflex time confirmed a mTBI injury at P35 and/or P70. Cognitive, psychosocial, and sensorimotor function was assessed over 1-week post-injury. Injured groups performed similarly to sham controls across all tasks. Immunofluorescence staining at 1 week detected an increase in glial activation markers in Sham + P70 brains only. Strikingly, 63% of Sham + P70 mice exhibited a skull fracture at impact, compared to 13% of P35 + P70 mice. Micro computed tomography of parietal skull bones found that a mTBI at P35 resulted in increased bone volume and strength, which may account for the difference in fracture incidence. In summary, a single mTBI to the adolescent mouse brain did not exacerbate the cerebral effects of a subsequent mTBI in adulthood. However, the head impact at P35 induced significant changes in skull bone structure and integrity. These novel findings support future investigation into the consequences of mTBI on skull bone.

## Introduction

Mild traumatic brain injuries (mTBI) during sports and recreational activities are a common injury in children and adolescents, with an estimated 1.1–1.9 million cases occurring annually in the US (1). Adolescents appear to have a particularly high incidence of mTBI (2). In high school students, mTBI accounts for 5–13% of all sporting injuries (3–5). In addition to a high incidence, evidence suggests that the adolescent brain may be particularly sensitive to the consequences of a mTBI compared to injuries sustained during childhood or adulthood (6–9). Disruption of key developmental processes ongoing during adolescence are hypothesized to be responsible for prolonged symptoms often observed after brain injury at this age compared to adults (10, 11).

For many professional and amateur athletes, exposure to mTBIs is a recurring event, and repetitive mTBIs are associated with poorer outcomes. Repeated mTBIs are prevalent in many collision sports (12), and the risk of a TBI is reportedly up to six times higher in an individual with a history of head injury compared to someone with no history (13). In addition, a study of over 6,000 high school athletes found that those who suffered two or more mTBIs reported more physical and cognitive symptoms compared to those with only one mTBI (14). Indeed, a second injury while still recovering from the first impact is thought to exacerbate the effect of the second hit (15, 16), perhaps as a consequence of ongoing metabolic dysfunction post-injury (17). In animal models, repetitive mTBIs have predominantly been shown to result in worsened outcomes including increased neuropathology and the protracted persistence of neurocognitive and psychosocial deficits (18–20) [as reviewed by Semple et al. (21)]. Conversely, some intriguing studies have conversely detected a degree of neuroprotection or preconditioning afforded by a prior head impact (22, 23).

While it is now generally accepted that multiple mTBIs in adult athletes can have cumulative, detrimental effects on brain structure and function, the threshold at which these consequences occur remains unclear. The time interval between successive impacts appears to be an important determinant of outcome (16, 24), although severity, age-at-insult, and the number of repeated injuries probably also play a role. Importantly, it is likely that many professional and amateur athletes who are exposed to a mTBI as adults, were also exposed to mTBI during adolescence *via* participation in sports and recreational activities during their youth (25). For example, an estimated 70% of all football players in the United States are under the age of 14, and football has the highest rate of sports-related mTBI among high school athletes (26, 27). A retrospective study of former NFL players aged 40–69 found that participation in tackle football prior to the age of 12 (and potential exposure to mTBI at this time) was associated with greater later-life cognitive impairments (28–30), and alterations in gray and white matter structures (28, 29, 31). While methodological limitations have been raised regarding these studies (32), whether a history of mTBI during childhood or adolescence influences an individual’s vulnerability to later-life brain injuries is a clinically and socially important question that has yet to be systematically investigated. Such issues can be challenging to address in patient populations, where the interval between early exposure to mTBI and later-life impairments may be many years. Further, a causal relationship cannot be established with cross-sectional patient studies (33).

Therefore, in the current study, we utilized a clinically relevant mouse model of mTBI, mild weight drop (mTBI), to evaluate the effect of a mTBI during adolescence on a subsequent mTBI at adulthood. Neurobehavioral and neuropathological outcomes were assessed, to test the hypothesis that mice receiving a mTBI at both adolescence and adulthood would have poorer neurological outcomes compared to uninjured mice or those with a mTBI at either age only.

## Materials and Methods

### Animals

**Figure 1:**
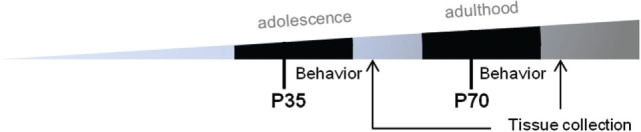
Experimental timeline. Prior to surgery at P35 or P70, mice underwent water maze training for five consecutive days. Mice then received either sham injury or mild traumatic brain injuries at P35 (adolescence) and/or P70 (adulthood), followed by behavioral assessments over a 7-day period. At 7 days, mice were euthanized and brains were collected for immunofluorescence.

A total of 94 C57Bl/6 male mice, sourced from the Walter and Elisa Hall Institute, Melbourne, were used in this study. Mice were provided with *ad libitum* access to food and water, and maintained under 12:12 h light/dark conditions, in groups of 4–5 before and after surgery, then individually housed during behavioral testing. All procedures were performed in accordance with the guidelines of the Australian Code of Practice for the Care and Use of Animals for Scientific Purposes from the Australian National Health and Medical Research Council, and were approved by the Florey Institute of Neuroscience and Mental Health Animal Ethics Committee (#15-052-UM). Prior to each surgery, mice were randomly assigned to mTBI or sham procedures using a random number generator.^1^ This created a total of six groups: (1) sham only at P35 (‘Sham’); (2) mTBI only at P35 (‘P35’); (3) sham at both P35 and P70 (‘Sham + Sham’); (4) sham at P35 then mTBI at P70 (‘Sham + P70’); (5) mTBI at P35 then sham at P70 (‘P35 + sham’); or (6) mTBI at both P35 and P70 (‘P35 + P70’) (Table 1). Each mouse in groups 3–6, therefore, received two doses of anesthesia and two instances of surgical preparation, once at P35 and again at P70. The timeline for experimental procedures is depicted in Figure 1. Mice underwent one bout of behavioral testing each; only the first two groups were tested over 1 week after P35 surgery, while all other groups were tested following P70. P35 was considered to reflect adolescence in mice, based on changes in synaptic plasticity, behavior, and sexual maturity around this time which parallels what is observed in human adolescence (34, 35). P70 was considered as young adult.

**Table 1:** Experimental groups.

Groups	At P35	At P70	*n*	Skull fracture at P70 injury	Terminal time point	Parietal bone analyzed^b^
‘Sham’	Sham	–	15	–	P42	Yes
‘P35’	mTBI	–	15	–	P42	Yes
‘Sham + Sham’	Sham	Sham	15	–	P77	Yes
‘P35 + Sham’	mTBI	Sham	15	–	P77	Yes
‘Sham + P70’	Sham	mTBI	19	12 (63%)^a^	P77	–
‘P35 + P70’	mTBI	mTBI	15	2 (13%)	P77	–

*^a^The number of fractures was significantly different between P70 only and P35 + P70 injured groups. p = 0.0051, Fisher’s exact test*.

*^b^The parietal bone plate, ipsilateral to the impact site, was collected ipsilateral to the impact site at 1 week or 6 weeks after mTBI or sham injury at P35, to examine the effect of P35 mTBI on bone integrity over time*.

### Mild Weight Drop (mTBI) Model

**Figure 2:**
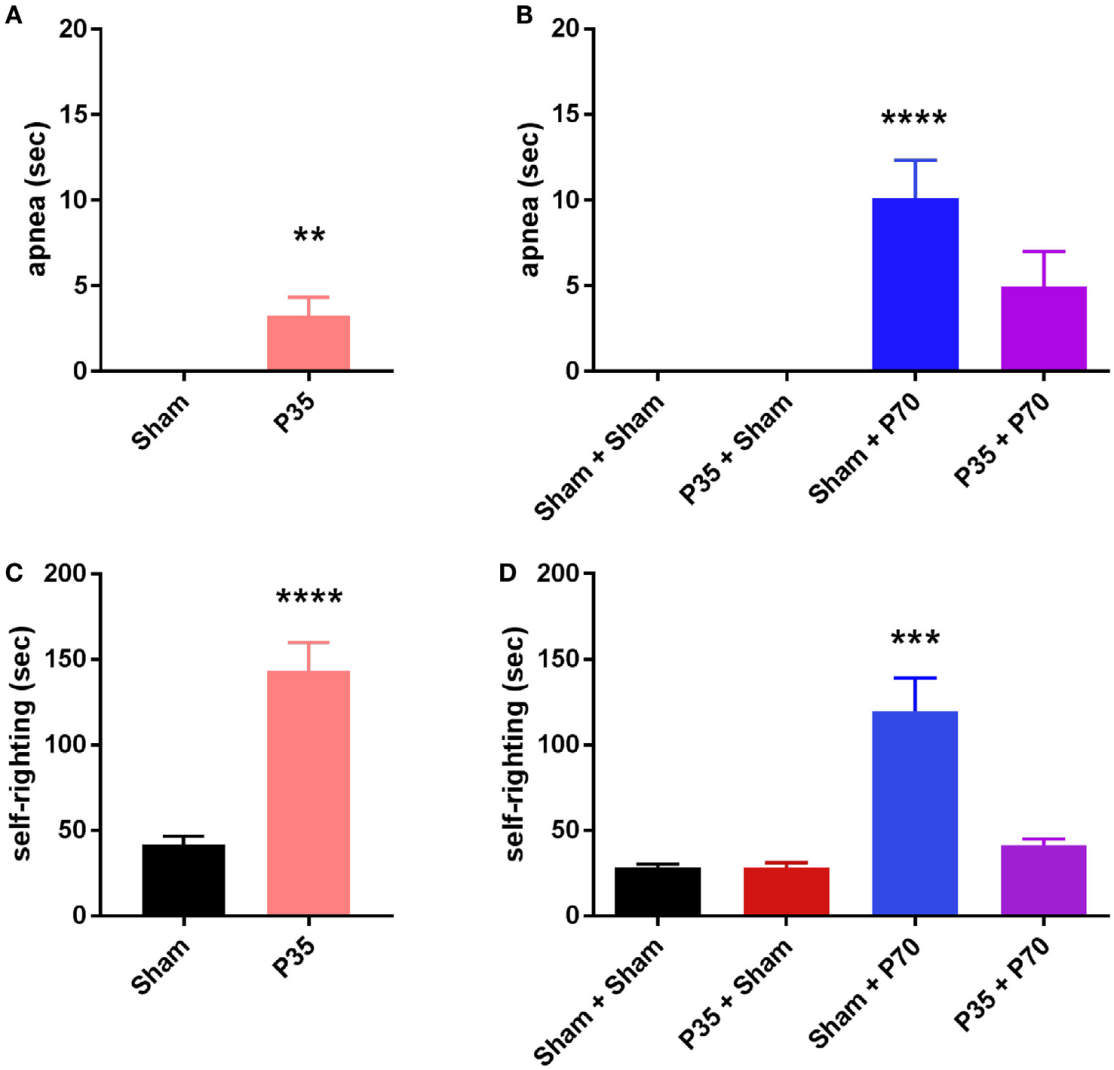
Length of apnea and latency to self-right following sham or mild traumatic brain injuries at P35 and/or P70. Panels **(A,C)** were assessed after injury at P35; **(B,D)** were assessed after injury at P70. **p < 0.01, ****p < 0.0001 compared to sham groups **(A)**, or compared to all other groups **(D)**; ^#^*p* > 0.05 (n.s.) versus all other groups. All injured mice exhibited a period of apnea and increased latency to self-right immediately post-injury compared to sham controls. Mice injured at P70 only (sham + P70) showed a delayed self-righting response compared to all other groups, including P35 + P70 injured mice.

The weight drop model was performed as previously described in detail (36). This commonly used model of mTBI reproduces a clinically relevant, blunt head trauma, consistent with a large proportion of mTBI in patients. Mice were anesthetized with 4% isoflurane in an induction chamber, then maintained at 2% isoflurane *via* nose cone during surgical preparation—a longitudinal incision of the scalp to expose the skull, and s.c. administration of the analgesic buprenorphine (0.05 mg/kg Temgesic^®^, Reckitt Benckiser). Ninety seconds after the induction of isoflurane, the mouse was removed from the nose cone and positioned on a stable surface under the weight drop apparatus, for alignment of the impactor rod with the center of the left parietal bone, midway between Bregma and Lambda and lateral of the sagittal suture. The 333 g impactor rod fitted with a 3 mm diameter tip was then raised to 1.5 cm (for mTBI at P35) or 2 cm (for mTBI at P70), and released. The release height was scaled according to age at the time of impact, determined from pilot experiments (Data S1 in Supplementary Material), to achieve a comparable injury severity at both time points based upon acute injury measures (e.g., self-righting time; Figures 2C,D). Sham-injured mice underwent identical surgical preparation and timing under anesthesia, but did not receive the actual impact. Following mTBI or sham injury, acute injury measures of apnea (latency until normal respiration resumed) and latency to self-right from a supine position were recorded (37). The skull was examined for evidence of a visible fracture, defined as a break or depression in the skull. Mice were de-identified and randomized to a numbered code to blind investigators during all subsequent analysis. One hour following injury/sham at P35 and P70, the Neurological Severity Score (NSS) was evaluated. The NSS is a broad assessment of general neurological function, including reflexes, balance, and locomotion, and involves the generation of a composite score out of 10, with 10 indicating greatest impairment and 0 indicating no impairment (36). Body weights were recorded throughout the study as a measure of general health; however, no differences were observed between the groups (data not shown).

### Behavioral Testing

In the week following sham or mTBI surgeries at P35 or P70, a battery of behavioral tests was used to assess cognitive, psychosocial, and sensorimotor functioning (*n* = 15–19/group). First, a water maze was used to assess whether mTBI impacted hippocampal spatial memory. An overhead camera tracked the animal’s swimming trajectory, for analysis using Ethovision 3.0.15 tracking software (Noldus, SDR Scientific). A 1.63 m diameter pool, filled with opaque water (21–23°C) was used. Cued task training was conducted for 5 concurrent days prior to the surgery date (i.e., P28–33, or P64–P68), across two daily blocks of three trials each per day. Each trial ended when the mouse located the hidden platform, located 0.5 cm below the water surface, or when 60 s had elapsed, at which time the experimenter would guide the mouse to the platform, with time to find the platform as the primary outcome measure. Three trial starting locations (North, South, and East relative to a West-located platform), were used in a random order to prevent successive starts from the same location. On day 1 post-surgery, the “test” consisted of six trials with the platform hidden (consistent with the training phase), followed by three trials with the platform raised and visible, the latter task being to evaluate swim speed (velocity) to check for potentially confounding visual and/or motor deficits. This protocol, with training prior to the injury, was chosen to provide an indication of spatial memory/recall during the acute post-injury phase, while also allowing sufficient time to also test for a range of other behavioral outcomes within the first week post-injury. A similar design has successfully been employed by others to evaluate retrograde amnesia after chemical and mechanical injuries to the hippocampus and entorhinal cortex (38, 39).

An accelerating rotarod (Harvard Apparatus, Holliston, USA) was used to test sensorimotor function on days 2 and 3 after surgery (40). The rotarod accelerated from 4 to 40 rpm over a 5 min period, and the trial was terminated when the mouse fell from the rod. Performance on three consecutive trials was averaged per mouse, per day. A ledged beam task was also used to assess motor ability on day 2 after surgery (41). This involved placing the mouse on the widest end of a tapered beam on a 30° incline, and video-recording as they traversed the beam toward a darkened cage. Performance was evaluated over a 60 cm section of the beam, and an error was defined as a weight bearing step onto the lower ledges of the beam. Errors were averaged from three consecutive trials per mouse.

An elevated plus maze (EPM; San Diego Instruments, USA) was used to test anxiety-like behavior on day 3 after surgery (42). A 10 min trial period was recorded by an overhead video camera, and percent time spent in the open arms relative to closed arms of the maze was quantified using Ethovision tracking software. Social interactions were evaluated in a 36 cm^2^ open field arena, on day 4 after surgery (43). In this test, experimental mice were allowed a 10 min period of solitary exploration for habituation to the arena, then a naive stranger mouse (sex, strain, and age-matched) was introduced. Social contact between the two mice (defined as being within 2 cm proximity) was quantified from video recordings.

### Immunofluorescence

Immunofluorescence staining was performed on a subset of mice (*n* = 5/group) to evaluate glial activation and axonal injury after mTBI. Mice were anesthetized and transcardially perfused with 4% paraformaldehyde, followed by overnight incubation of collected brains in the fixative. Brains were transferred to a 30% sucrose solution for 3–5 days, then embedded in Optimal Cutting Temperature solution (Sakura Finetek USA Inc.) and frozen at −80°C. 12 µm thick coronal sections were collected spanning the parietal lobes. Five sections, evenly spaced between Bregma 0.0 to −3.0 mm, were stained for quantification and averaged per brain. Frozen brain sections were thawed and rehydrated, then incubated in blocking solution containing 10% normal donkey serum and 0.1% Triton-X-100 in phosphate buffered saline (PBS). Primary antibodies to detect glial fibrillary acidic protein (rabbit polyclonal GFAP, DAKO; 1:100), ionized calcium binding adaptor molecule 1 (goat polyclonal Iba-1, Abcam; 1:750), and beta amyloid precursor protein (rabbit polyclonal βAPP, Invitrogen; 1:750) were applied for overnight incubation at 4°C. For GFAP and Iba-1, secondary antibodies were applied the following day during a 1 h incubation period (1:250; donkey anti-rabbit AF 594 and donkey anti-goat AF 488; Molecular Probes™, ThermoFisher Scientific), followed by treatment with 4′,6-diamidino-2-phenylindole (DAPI) as a nuclear counter-stain. For βAPP, the secondary antibody (1:300; biotinylated goat anti-rabbit IgG, Vector Laboratories) was followed by incubation with an avidin–biotin complex (Vector Laboratories) for 30 min, then incubation with diaminobenzidine for 2 min for color development.

Photomicrographs were captured of GFAP and Iba-1 stained sections at 10× magnification, using a Carl Zeiss Axioplan-2 fluorescence microscope, under fixed exposure times (140 ms and 850 ms, for GFAP and Iba-1, respectively). Staining intensity was quantified using ImageJ (National Institutes of Health, USA) (44).^2^ In brief, images were converted to 8 bit and thresholded, with analysis restricted to a 750 µm × 750 µm region of interest centered on the cortex and hippocampus of both hemispheres (i.e., four images per section), under the impact site at the level of the dorsal hippocampus (45, 46). For βAPP, photomicrographs of βAPP stained slides were captured at 10× magnification on a Olympus BX51 microscope, of the corpus callosum at the midline, as well as ipsilateral and contralateral external capsules. Qualitative assessment was performed on five sections per brain, spanning the anticipated injury site (sections adjacent to those used for GFAP and Iba1 staining). A positive control was run alongside the experimental tissue and exhibited positive staining with the anticipated morphology of elongated axonal-like strands and retraction bulbs (47), indicating that the staining was successful.

### Bone Analysis

A disparity in the number of fractures after P70 injury between the Sham + P70 group and the P35 + P70 group (Table 1) suggested that mWD injury at P35 may have affected the skull response to a subsequent injury. Therefore, we compared the ipsilateral parietal skull bone plates from a subset of mice (*n* = 5/group) that received sham or mWD at P35, collected either 1 week after P35 injury (i.e., at P42) or 6 weeks after P35 injury (i.e., at P77; following sham surgery at P70). After collection, bones were fixed overnight in 4% paraformaldehyde, then stored in 70% ethanol at 4°C until scanning. Microcomputed tomography (µCT) was used to evaluate bone structure and integrity, using a Skyscan1076 µCT (Bruker, Kontich, Belgium) at St. Vincent’s Institute of Medical Research, Melbourne (voxel resolution 9 µm; aluminum filter AI 0.5 mm; source voltage 49 kV; source current 212 µA; exposure 2,400 ms; rotation 0.5° across 180°; frame averaging of 1) (48, 49). Images were reconstructed using NRecon (Bruker; Version 1.6.3.1) with the following parameters: CS to image conversion 0.0–0.11; ring artifact 6; pixel defect mask 5%; and beam hardening correction 35%. Following reconstruction, the region of interest for each bone was determined using CTAn (Bruker; Version 1.11.8.0) as a 3 mm × 3 mm square centered on the site of impact. In brief, images were separated into 255 grayscale values (0–255, where 0 = black and 255 =white). Thresholds used for quantification of bone structural parameters, whereby 59–255 represented bone, were determined using the automatic “otsu” algorithm within CTAn and visual inspection of representative images. 2D and 3D data were generated for all analyses, the latter by using the “marching cubes” algorithm from thresholded data in CTAn. Bone volume, bone volume fraction (% bone volume/tissue volume), and mean polar moment of inertia were quantified. “Tissue” (i.e., not bone) refers to non-mineralized tissue which was not detected by μCT, and likely includes marrow, vasculature, and cartilage.

### Statistical Analysis

All statistical analyses were performed using Prism GraphPad v. 7.0 (Graphpad, La Jolla, CA, USA). The sham + P70 group was first analyzed as a whole for comparison to the other groups, and then reanalyzed as fracture or non-fracture subgroups. One or two-way analysis of variance (ANOVA) tests was used for behavioral and immunofluorescence data, with repeated measures when appropriate (e.g., for water training across time), and using *post hoc* analyses to compare between specific groups when significant main effects were detected. A two-tailed Fisher’s exact test was used to compare the rate of fractures between two groups, and unpaired *t*-tests compared between sham and P35 groups for acute injury measures and μCT data. Statistical significance was set at *p* ≤ 0.05, and all data presented as mean ± SEM.

## Results

### Acute Responses to mTBI at P35 and/or P70

At the time of P35 (adolescent) surgeries, mTBI was confirmed by observation of acute injury measures in P35 injured mice compared to sham controls (Figure 2). P35 mice exhibited a period of apnea immediately post-injury (unpaired *t*-test, *t*_28_ = 3.04, *p* = 0.0049), and an increased latency to self-right compared to sham mice (*t*_28_ = 5.95, *p* < 0.0001).

At the time of P70 (adulthood) surgeries, Sham + P70 mice showed a significantly longer period of apnea (one-way ANOVA, *F*_3,60_ = 9.16, *p* < 0.0001), compared to both Sham + Sham and P35 + Sham groups (Tukey’s *post hoc p* < 0.001). The latency to self-righting post-injury was longer for Sham + P70 mice compared to all other groups, including P35 + P70 mice (one-way ANOVA, *F*_3,60_ = 14.78, *p* < 0.0001; Tukey’s *post hoc p* < 0.0001).

In contrast, the NSS assessment failed to detect any significant neurological impairments at 1 h post-injury, at either P35 or P70 (Data S2 in Supplementary Material).

Of note, at P70, we observed that 12 of the 19 mice (63%) in the Sham + P70 group experienced an evident mTBI-induced skull fracture directly under the impact site (63%), compared to 2 of 15 (13%) of the P35 + P70 group (Fisher’s exact test, *p* = 0.0051). The fractures were typically linear in nature, 2–3 mm in length corresponding to the impactor tip diameter. As the presence of a fracture could be a confounding factor, the Sham + P70 group was sub-divided into fracture (*n* = 12) versus no-fracture (*n* = 7) groups for direct comparison. The presence of a fracture had no effect on apnea duration (*t*_17_ = 1.06, *p* = 0.3037) but appeared to non-significantly increase self-righting time (*t*_17_ = 1.9, *p* = 0.0743; no fracture: 74 ± 19 s; fracture: 146 ± 27 s).

### Neurobehavioral Outcomes Following P35 Injury

**Figure 3:**
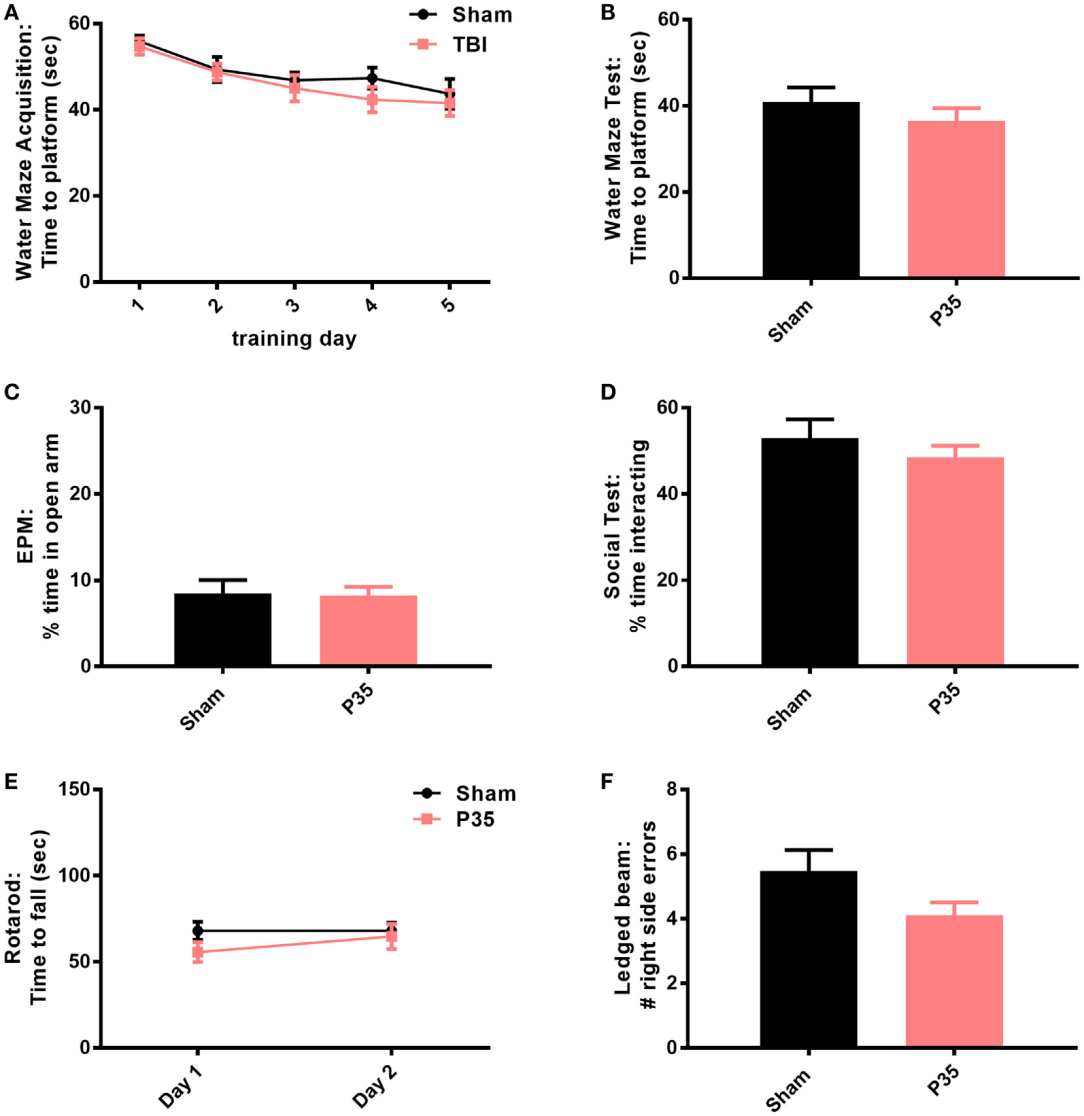
Behavioral outcomes after mild traumatic brain injuries (mTBI) or sham injury at adolescence. All mice were trained in a water maze for 5 days prior to sham or mTBI injury at P35 **(A)**. On days 1–6 post-injury, mice were tested in a battery of behavioral tasks, including an additional water maze session **(B)**, elevated plus maze (EPM) **(C)**, social interactions **(D)**, rotarod **(E)**, and ledged beam task **(F)**. None of the groups differed from each other in any of the tests (*p* > 0.05).

Cognitive, psychosocial, and sensorimotor function were first evaluated over the first week following mTBI or sham injury at adolescence, to evaluate the impact of a mTBI at this age (Figure 3). Mice were trained in the water maze for 1 week prior to injury at P35. As expected, prior to injury at P35, sham and P35 groups did not differ in their ability to learn this task over time (two-way RM ANOVA, effect of injury: *F*_1,28_ = 0.81, *p* = 0.3762; effect of time: *F*_4,112_ = 10.37, *p* < 0.0001; interaction: *F*_4,112_ = 0.31, *p* = 0.8723). On day 1 post-injury, both P35 injured mice and sham controls showed comparable memory retention of the task (unpaired *t*-test, *t*_30_ = 0.058, *p* = 0.3457). Similarly, no differences were detected between injury groups in the EPM, a test for anxiety (*t*_30_ = 0.16, *p* = 0.8772), nor was their social interest in an unfamiliar stimulus mouse (*t*_30_ = 0.87, *p* = 0.3904). Finally, mTBI did not influence sensorimotor performance on the rotarod (two-way RM ANOVA, effect of time *F*_1,28_ = 0.93, *p* = 0.3427; effect of injury *F*_1,28_ = 1.35, *p* = 0.2557; interaction: *F*_1,28_ = 0.93, *p* = 0.3427), or the ledged beam task (*t*_30_ = 1.80, *p* = 0.0813).

### Behavioral Outcomes Following P70 Injury

**Figure 4:**
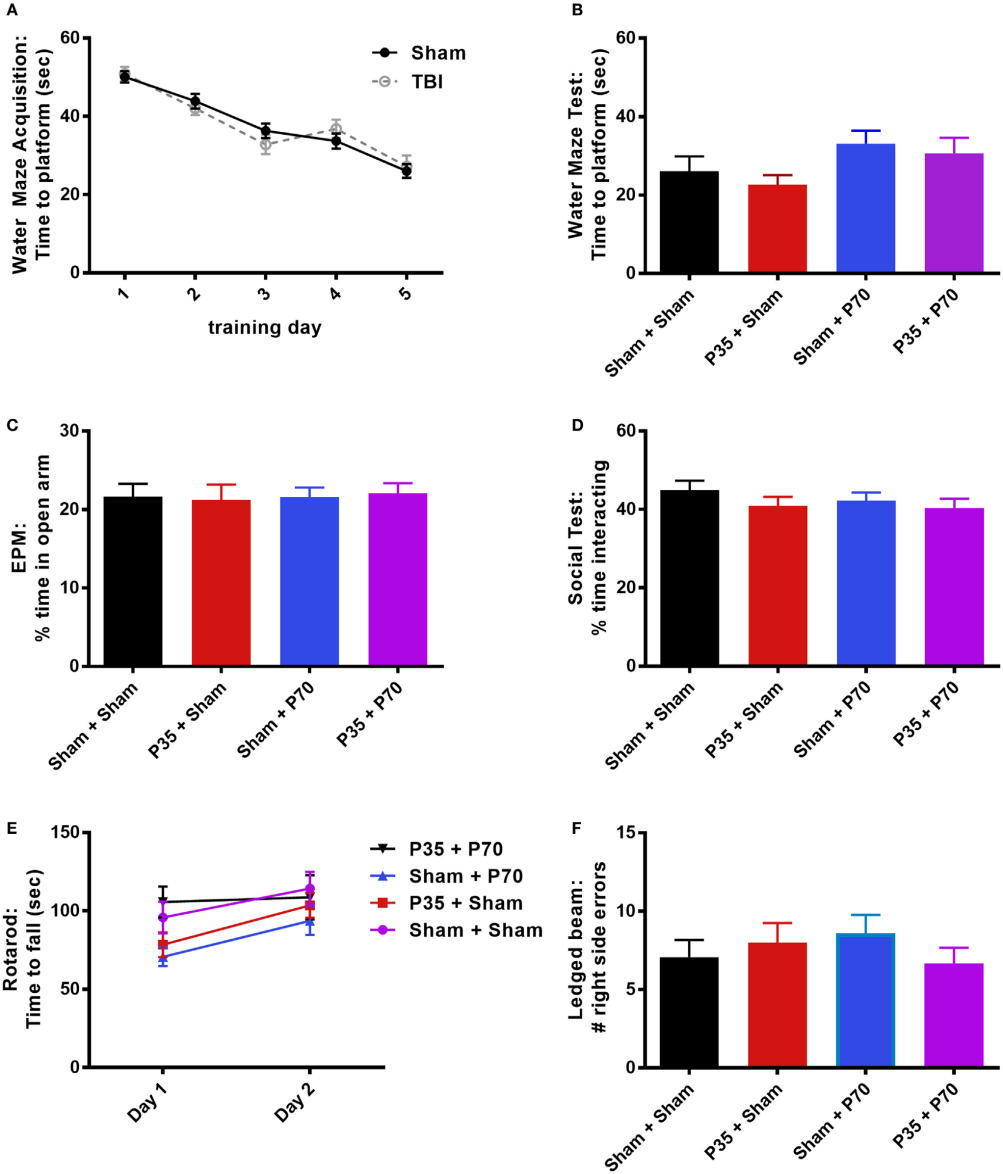
Behavioral outcomes after mild traumatic brain injuries (mTBI) or sham injury at adulthood. Mice that received mTBI or sham at P35 were trained in a water maze for 5 days prior to P70 injury **(A)**. On days 1–6 post-injury, mice were tested in a battery of behavioral tasks, including an additional water maze session **(B)**, elevated plus maze (EPM) **(C)**, social interactions **(D)**, rotarod **(E)**, and ledged beam task **(F)**. None of the groups differed from each other in any of the tests (*p* > 0.05).

Cognitive, psychosocial, and sensorimotor function were next evaluated over the first week following mTBI or sham injury at adulthood, to evaluate the impact of a mTBI at adulthood and determine whether a prior insult during adolescence would influence outcomes (Figure 4). All mice were first trained in the water maze for 1 week prior to injury at P70. The presence of a prior mTBI at P35 did not influence their ability to learn this task over time (two-way RM ANOVA, effect of injury: *F*_1,63_ = 0.39, *p* = 0.5334; effect of time: *F*_4,252_ = 64.26, *p* < 0.0001; interaction: *F*_4,252_ = 0.64, *p* = 0.6327). One day after mTBI or sham injury at P70, a final water maze session did not detect any differences between the groups (one-way ANOVA, *F*_3,60_ = 1.88, *p* = 0.1422), indicating that mTBI exposure did not impair hippocampal-dependent memory of a previously learned platform location. Consistent with a lack of cognitive deficits, no differences were detected between injury groups in tests for anxiety (EPM; one-way ANOVA, *F*_3,60_ = 0.05, *p* = 0.9863), social interactions (one-way ANOVA, *F*_3,57_ = 0.79, *p* = 0.5051), rotarod performance (two-way RM ANOVA, effect of time *F*_1,60_ = 11.60, *p* = 0.0012; effect of injury *F*_3,60_ = 2.28, *p* = 0.0882; interaction: *F*_3,60_ = 0.93, *p* = 0.4322), or balance in the ledged beam task (one-way ANOVA, *F*_3,60_ = 0.59, *p* = 0.6228).

Direct comparison of Sham + P70 mice versus without a skull fracture did not detect any significant differences between the subgroups for any behavioral test (*p* > 0.05).

### Glial Reactivity Following mTBI at P35 and/or P70

**Figure 5:**
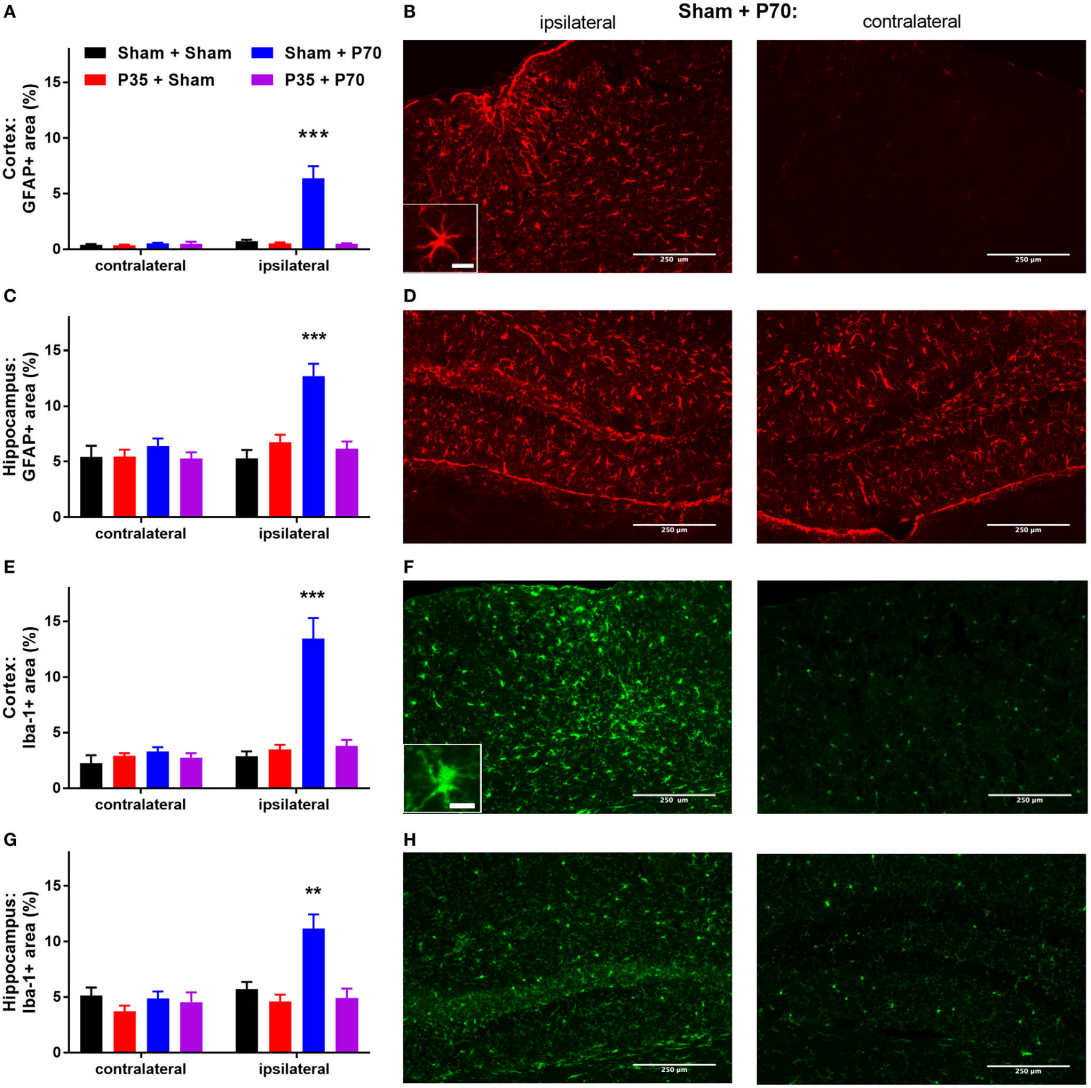
Glial reactivity after mild traumatic brain injuries (mTBI) or sham injury at P35 and/or P70. Glial reactivity was quantified from immunofluorescence at 7 days after injury at P70. **(A,B)** GFAP in the cortex, and **(C,D)** hippocampus; **(E,F)** Iba-1 in the cortex, and **(G,H)** hippocampus. Quantification and representative images from the sham + P70 group are presented. Scale bar = 250 µm (inset = 15 µm).

Immunofluorescence staining of GFAP and Iba-1 was quantified at 7 days post-injury, as an indicator of astrocytic and microglial reactivity, respectively. After injury at P35, no differences in GFAP or Iba-1 fluorescence intensity were observed in the cortex or hippocampus of mTBI mice compared to sham controls (*p* > 0.05). In contrast, after injury at P70, Sham + P70 injured mice showed a 12-fold increase in GFAP immunofluorescence in the ipsilateral cortex compared to the contralateral cortex (two-way ANOVA: effect of injury: *F*_3,19_ = 21.4, *p* < 0.0001; effect of location: *F*_1,19_ = 20.74, *p* < 0.0001; interaction: *F*_3,19_ = 20.03, *p* < 0.0001; Tukey’s *post hoc, p* < 0.0001; Figures 5A–D). GFAP reactivity in the ipsilateral cortex of Sham + P70 mice was also significantly higher than in the ipsilateral cortex of all other groups (Tukey’s *post hoc, p* < 0.0001). Similarly, GFAP reactivity in ipsilateral hippocampus of Sham + P70 mice was significantly higher than contralateral, and all other groups (two-way ANOVA: effect of injury: *F*_3,19_ = 21.40, *p* < 0.0001; effect of location: *F*_1,19_ = 13.44, *p* = 0.0003, interaction: *F*_3,19_ = 7.04, *p* = 0.0002; *post hoc, p* < 0.001). Sub-group analysis to compared Sham + P70 mice versus those without a skull fracture found that both groups consistently showed increased GFAP reactivity in the ipsilateral cortex and hippocampus compared to the contralateral side, indicating that a fracture at the time of impact did not account for these findings.

The pattern of Iba-1 immunofluorescence mirrored that of GFAP (Figures 5E–H). In the cortex, the ipsilateral cortex of Sham + P70 mice showed a fourfold increase in Iba-1 reactivity compared to the contralateral cortex, and was also significantly higher than all other groups (two-way ANOVA, effect of injury: *F*_3,19_ = 18.20, *p* < 0.0001; effect of location: *F*_1,19_ = 20.17, *p* < 0.0001); interaction (*F*_3,19_ = 13.31, *p* < 0.0001; Tukey’s *post hoc, p* < 0.0001). Similarly, Iba-1 reactivity in the ipsilateral hippocampus of Sham + P70 mice was elevated compared to the contralateral side and all other groups (two-way ANOVA, effect of injury: *F*_3,19_ = 8.48, *p* < 0.0001; effect of location: *F*_1,19_ = 9.93, *p* = 0.0019; interaction: *F*_3,19_ = 5.81, *p* = 0.0008; Tukey’s *post hoc, p* < 0.01). Sub-group analysis to compared Sham + P70 mice versus those without a skull fracture found that the presence of a fracture did influence Iba-1 reactivity. Sham + P70 mice with a fracture showed significantly higher Iba-1 in the ipsilateral cortex and hippocampus compared to their respective contralateral regions (*t*-tests, *p* < 0.0001), whereas Sham + P70 mice without a fracture did not reach statistical significance (*t*-test, *p* = 0.0632 and 0.0951, for cortex and hippocampus, respectively).

**Figure 6:**
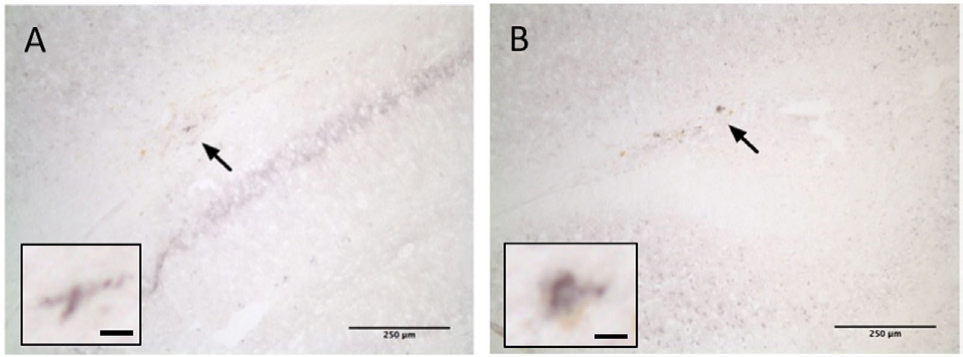
βAPP staining at 7 days after injury. One brain from the P70 group exhibited positive βAPP staining in the corpus callosum ipsilateral to the injury (arrow). Two examples are shown **(A,B)**. Scale bar = 250 µm (inset: 15 µm).

Examination of brain tissue stained for βAPP failed to detect any positive staining in sham, P35, or P35 + P70 groups. One brain from the P70 group exhibited a small degree of positive staining on sections collected immediately under the impact site in the corpus callosum/external capsule, indicating mild axonal damage (Figure 6). This very limited amount of positive βAPP staining suggests that axonal damage was uncommon in this model, reiterating the very mild nature of the impact.

### Bone Analysis

**Figure 7:**
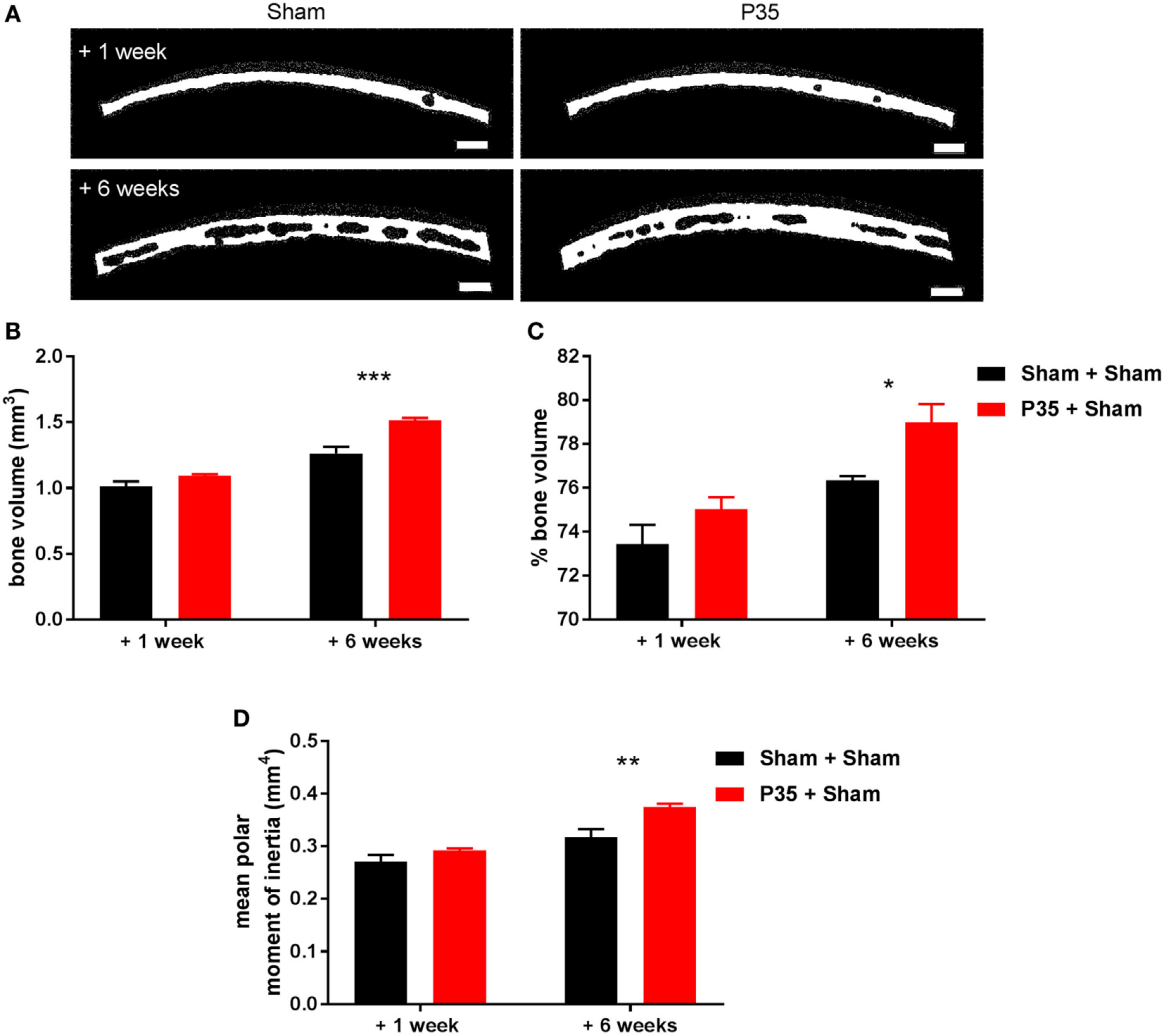
Parietal bone analysis. Parietal bone ipsilateral to the impact site was collected either 1 or 6 weeks after sham or mild traumatic brain injuries (mTBI) at P35. Representative images of reconstructed bones **(A)** highlight differences in bone volume dependent on age and the presence/absence of a mTBI at P35. Scale bar = 250 µm. Quantification of bone volume **(B)**, % bone volume **(C)**, and mean polar moment of inertia **(D)** found that a mTBI at P35 was sufficient to alter parietal skull structure and integrity at adulthood (+6 weeks). *n* = 5/group. *p < 0.05, **p < 0.01, ***p < 0.001.

Based on our observation that 63% of the Sham + P70 group experienced a skull fracture at the time of P70 mTBI compared to 13% of the P35 + P70 group (Table 1), we sought to evaluate the effect of mTBI at P35 on subsequent bone structure and integrity. To do this, the parietal bone plate, ipsilateral to the impact site, was collected from Sham and P35 injured mice at 1 week after the P35 injury (i.e., P42), and from Sham + Sham and P35 + Sham groups at 1 week after the P70 injury (i.e., 6 weeks after P35 sham/injury, at P77) (Figure 7). From 3D reconstructions, μCT analysis detected a significant increase in parietal bone volume with increasing age of the animals (two-way ANOVA effect of time, *F*_1,14_ = 116.9, *p* < 0.0001), as well as an effect of P35 injury (*F*_1,14_ = 28.1, *p* < 0.0001), and injury*time interaction (*F*_1,14_ = 7.68, *p* = 0.0150). Specifically, at 6 weeks, mice that received a mTBI at P35 had a significantly higher bone volume compared to sham controls (*p* < 0.001). Similar findings were observed with regards to bone volume fraction (bone volume/tissue volume) (two-way ANOVA effect of time, *F*_1,14_ = 24.73, *p* = 0.0002; effect of injury, *F*_1,14_ = 9.47, *p* = 0.0082; interaction, *F*_1,14_ = 0.61, *p* = 0.4472; *post hoc, p* < 0.05). Finally, the mean polar moment of inertia, an indicator of the bone’s resistance to torsion, also showed an increase over time with age (two-way ANOVA *F*_1,14_ = 43.92, *p* < 0.0001) and injury (*F*_1,14_ = 16.52, *p* = 0.0012; interaction, *F*_1,14_ = 3.39, *p* = 0.0870). The mean polar moment of inertia was significantly higher at 6 weeks in P35 injured mice (P35 + Sham) compared to sham only controls (*post hoc, p* < 0.01), indicating increased bone strength in this group. Together, these differences in skull properties likely accounts for the reduced incidence of fractures when P35 injured mice received a second impact at P70 (P35 + P70), compared to those who received only an injury at P70 (Sham + P70).

## Discussion

### Summary

The consequences of a mTBI in adolescence on a subsequent mTBI in adulthood were investigated in this study, to evaluate whether a prior injury in adolescence would result in more severe consequences to a second injury in adulthood. However, our findings did not support this hypothesis. Instead, mice that received a mTBI at adulthood only (Sham + P70) exhibited a significantly longer period of apnea and latency to self-right compared to sham animals, as well as compared to mice that received two mTBIs (P35 + P70). None of the injured groups showed detectable impairments on any tests for cognitive, psychosocial, or sensorimotor function across the first week post-injury, nor evidence of neurological impairment on the NSS acutely post-injury. At 1 week, only Sham + P70 mice showed a robust, significant elevation in GFAP and Iba-1 reactivity in the ipsilateral cortex and hippocampus, which was surprisingly not present in the P35 + P70 group. A higher incidence of skull fracture in the Sham + P70 group may have been a confounding factor; however, sub-group analysis found that even those without an overt skull fracture showed pronounced acute injury measures and glial reactivity compared to the other groups, indicating that fracture was not solely accountable for these findings. Instead, investigation of skull bone properties by μCT revealed that a mTBI at P35 resulted in the parietal bone having a greater volume and strength by adulthood, which may have influenced the consequences of a subsequent mTBI at this time. These intriguing findings highlight the need for further research into age-specific effects of mTBI, and a greater understanding of how skull bone responds to concussive and sub-concussive impacts.

### Differing Thresholds for Neuroinflammatory Versus Neurobehavioral Consequences of mTBI

We confirmed that a head impact had indeed occurred in the mTBI groups in this study, by quantification of acute injury measures—injured mice exhibited a period of apnea immediately following the impact, and a delayed self-righting response compared to sham-operated controls. This response was similar after mTBI at P35 or P70, suggesting that a comparable severity had been induced at these times. Somewhat surprisingly, mTBI failed to result in detectable impairments in any of the neurobehavioral domains examined, including general neurological, cognitive, sensorimotor, and psychosocial. This observation could be attributed to low sensitivity of these assessments to TBI-related deficits. The chosen tasks have previously been used to detect functional deficits in several other models of TBI, however, with sensitivity even for mild, concussive-like injuries (19, 20, 37, 50–52). Another possibility is that we did not examine the optimal time point for functional deficits after mTBI. Many symptoms of mTBI reportedly recover quickly, in both patients and adult animal models, typically resolving within 7–10 days (53). For example, a recent study by our group detected transient cognitive deficits in the Morris water maze only at 3 days after mTBI in adult rats, while more subtle molecular and structural changes were evident until at least 30 days after injury (54). Further, a recent study by Tagge and colleagues, who reported very acute (2 min post-injury) and transient neurobehavioral deficits after experimental mTBI, which had resolved by reassessment at 3 h post-injury (55). This suggests that, for a very mild insult, even the 1 h time point for evaluation of the NSS score may have missed some transient deficits resulting from the head impact. A third possibility is that our injuries were simply below the threshold required to induce detectable neurobehavioral consequences. Our model may, therefore, be better defined as a “sub-concussive” insult, whereby head impact results in acute dysfunction (delayed righting and apnea) but no overt neurological impairment by 1 h post-injury (NSS), nor functional deficits over the first week post-injury. While we acknowledge that not every head impact is necessarily pathological, and the definition of sub-concussion remain controversial as a head impact without the clinical symptoms of a concussion (56), our findings are still of strong interest as even such a minor mechanical impact resulted in changes to the skull bone (after impact at P35) and a neuroinflammatory response (after impact at P70 alone). Despite an increasing concern regarding the consequences of repeated sub-concussive injuries in athletes (57, 58), few experimental studies have focused on this paradigm, and additional studies are clearly warranted.

Despite the lack of behavioral deficits in this model, we did observe a sub-acute neuroinflammatory response at 1 week after mTBI at adulthood, specifically in mice that had also received a sham injury at P35 (i.e., Sham + P70 group). This time point, and the markers of interest, were chosen based on previous studies demonstrating that even a single mild head impact can induce glial reactivity and axonal injury, including proliferation and hypertrophy, for at least 10 days post-injury (59, 60). In both the cortex and hippocampus ipsilateral to the site of impact, an increase in GFAP and Iba-1 staining reflects an increase in reactive astrocytes and microglia/macrophages, respectively, as a result of cell proliferation, migration, and/or activation (61). This observation is consistent with recent studies from our group, using mTBI in adult mice or mild fluid percussion injury in adult rats, whereby localized glial reactivity was also observed in the absence of behavioral deficits (51, 62). These findings also align with an increasing number of reports of athletes with a history of concussive or sub-concussive injuries, who are clinically asymptomatic yet show persistent changes in functional connectivity or brain biochemistry (57, 58, 63). Together, our findings indicate that the threshold at which a mTBI results in glial reactivity is lower than the level of injury required for neurobehavioral deficits or pronounced axonal injury to manifest. Again, this finding is consistent with the recent work by Tagge and colleagues, who recently observed astrogliosis and microgliosis in the ipsilateral cortex from 3 days to at least 2 weeks after experimental mTBI in mice, despite the resolution of any neurobehavioral impairments by 3 h post-injury (55).

### Does an Adolescent mTBI Result in Poorer Outcomes After a Subsequent mTBI at Adulthood?

The lack of an exacerbated outcome for the P35 + P70 group in terms of neurobehavioral and neuropathological outcomes was surprising, contrary to our hypothesis and the majority of published findings to date in experimental mTBI models. The cumulative effects of repeated mTBI in terms of cognitive (18, 20, 64), sensorimotor (65), and psychosocial deficits (20), as well on the neuroinflammatory response (20, 59), have been well-characterized in adult animals, albeit typically with an inter-injury interval of hours to days (66).

Here, we instead chose to focus on the potential consequences of injuries occurring during two distinct developmental periods. The first injury was induced during adolescence, as this is a time when many young athletes, both amateur and professional, are first exposed to mTBIs through involvement in contact sports. Adolescence is a time of ongoing brain maturation and development (67), and several studies have suggested that adolescents have poorer outcomes after mTBIs compared to older athletes (6, 8, 9). By examining the effects of a mTBI during adolescence (P35) followed by a second injury at adulthood (P70), we employed a longer interval (5 weeks between injuries) compared to most previous repetitive mTBI studies. The time window between injuries is likely to be an important determinant of long-term consequences after repetitive mTBI, with a shorter window ensuring that a second injury occurs while the brain is still recovering from the first (16, 68, 69). The very mild nature of our injury model may also have been below the threshold necessary to induce cumulative consequences after a subsequent impact.

As the weight drop model has not previously been characterized in P35 mice, we first conducted a pilot study and chose the 1.5 cm drop height to induce mTBI as it produced apnea and a delayed righting reflex compared to sham controls. The lack of neurobehavioral deficits that one would expect to result from a mTBI does not negate the possibility that repeated impacts at this sub-concussive severity could result in cumulative effects after a second impact at a later time point. Increasing clinical evidence suggests that sub-concussive impacts, defined as a cranial impact that does not result in symptoms associated with concussion, may nevertheless have an adverse long-term effect in some individuals, particularly after repeated exposures (56, 70)

In contrast, we unexpectedly found that mice injured at both P35 + P70 showed a reduced inflammatory response compared to those injured at P70 only (Sham + P70 group). This finding raises the possibility that a head impact at P35 (i.e., adolescence) may have a neuroprotective or pre-conditioning effect on the response to a subsequent head impact at adulthood. This concept is supported by similar findings in a recent rat study, whereby a mTBI at P30 appeared to protect the brain against functional deficits following a second injury at P60 (23), as well as evidence that repetitive mTBIs prior to a severe TBI resulted in reduced motor deficits compared to animals with severe TBI alone (22). Taken together, it remains unclear whether this apparent beneficial effect of a prior head impact is a consequence of the effect on skull bone, or a phenomenon related to the response of the brain parenchyma itself. Future studies which both exclude or focus on the contribution of skull bone to repeated head impacts are now needed.

### Altered Skull Bone Properties After a mTBI at Adolescence

The finding that the Sham + P70 group showed a robust neuroinflammatory response compared to all other groups, including P35 + P70 mice, may have resulted from the large disparity in the frequency of skull fractures observed in the sham + P70 group at the time of the P70 mTBI (63% compared to only 13% of Sham + P70 mice; see Table 1). To consider the effect of this possible confound, we conducted additional statistical analyses of the Sham + P70 group, dividing them into those with versus without an apparent fracture. While a fracture clearly had a detrimental effect on latency to self-right and the extent of Iba-1 reactivity, other measures (e.g., the length of acute apnea immediately following injury, and the extent of GFAP reactivity), were not dependent on the presence of a fracture. Thus the increased rate of fractures within the Sham + P70 group does not solely explain the observed differences in acute injury measures and neuroinflammation.

Instead, we present the hypothesis that a head impact at adolescence resulted in some form of pre-conditioning, such that the brain and/or skull bone were more resistant to the consequences of a second impact sustained at P70 compared to those animals that had not received a prior injury. Based on the striking difference in skull fracture rate between these groups, whereby the P35 + P70 group had fewer fractures compared to Sham + P70 mice, we focused our attention on the parietal skull bone plate using µCT, comparing those that had an injury at P35 (P35 + sham) versus those that only received sham surgeries (sham + sham). One potential limitation of the study is that we did not collect bones from the other injury groups (i.e., those who had also received an impact at P70), so cannot speculate on the additional effect that this had on the calvarium.

The skull is comprised of internal and external layers of cortical bone separated by trabecular bone known as diploe. At P35 there appears to be little diploe between thin layers of compact bone, and an overall increase in bone volume is observed with increasing age when comparing bone samples collected at 1 week compared to 6 weeks after P35, consistent with a previous report in the developing rat (71).

**Figure 8:**
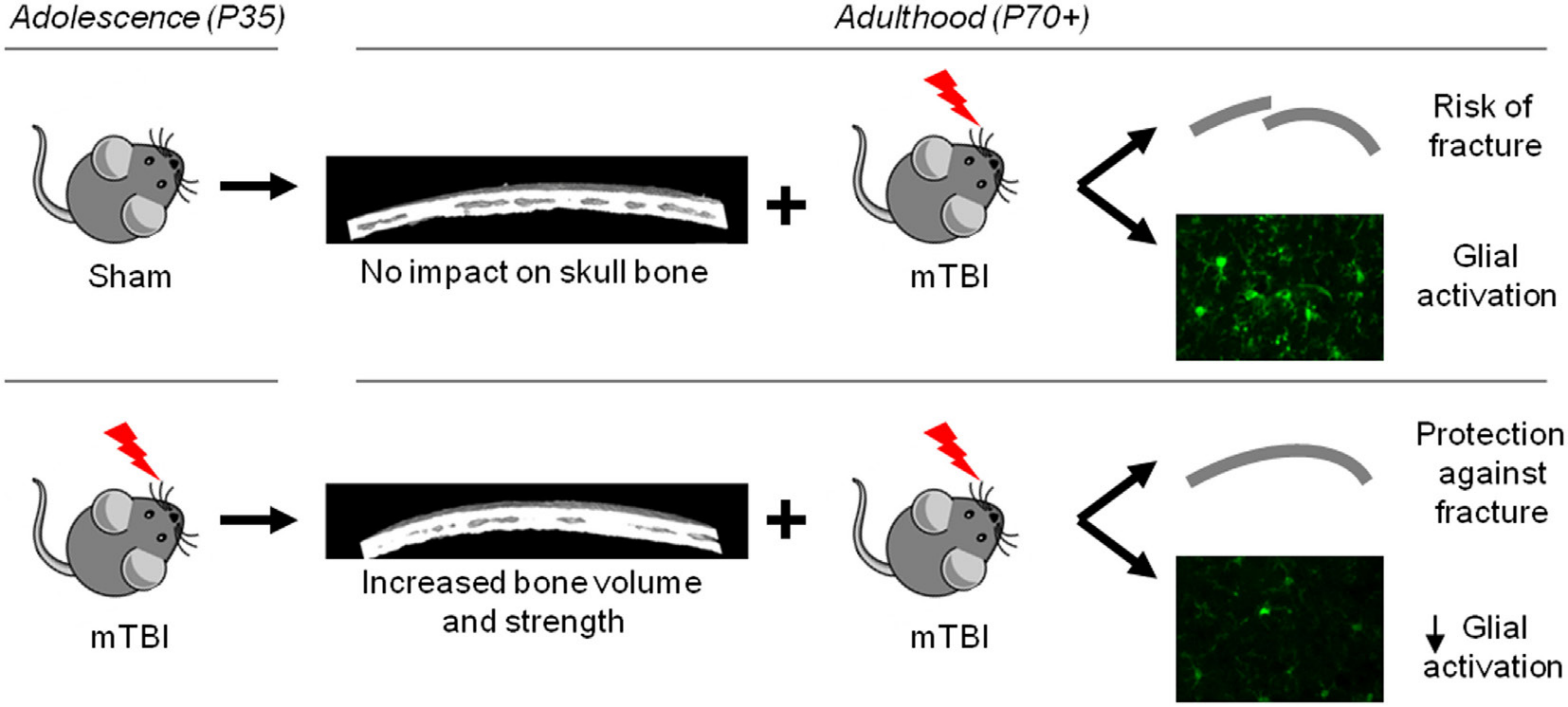
Schematic summary of study findings. Mild TBI at adolescence resulted in an increase in bone volume and strength by adulthood, which likely underlies the observed neuroprotection seen in mice that received a second mild traumatic brain injuries (mTBI) at adulthood (lower panel), compared to mice that received a mTBI at adulthood only (upper panel).

Independent of this age-dependent change, we observed striking changes in skull bone structure and integrity at 6 weeks after P35 mTBI, compared to age-matched sham controls, resulting in bone of increased volume and strength. These novel observations likely account for a resistance to skull fracture when mice previously exposed to a mTBI at P35 then sustain a second mTBI at P70. As bone thickness influences how a mechanical impact is transduced to the underlying tissue (72), we hypothesize that increased bone volume in the P35 + P70 group resulted in a reduced force of impact on the underlying cortical tissue, resulting in the reduced neuroinflammatory response observed in P35 + P70 compared to sham + P70 mice (Figure 8).

There are several potential mechanisms through which mTBI at P35 may have led to enhanced bone volume at the impact site. First, mTBI may have induced micro-fractures in the parietal bone, and thereby stimulated a localized reparative response featuring enhanced bone formation by osteoblasts (73). In addition, as mTBI was performed with the pericranium intact, direct impact to this tissue may have activated the osteogenic progenitor population of the inner cambium layer, triggering their proliferation and differentiation into osteoblasts (74). Osteogenic responses to periosteal trauma are well-characterized following long bone trauma (74, 75), but have also been observed in the skull following trauma-induced subperiosteal hematoma (76). Interestingly, the osteogenic cambial layer is at its thickest in the fetus and gradually becomes thinner with age (74), raising the possibility that trauma to the pericranium and underlying bone could induce a greater osteogenic response in juveniles than in adults. Indeed, the capacity for bone formation may be enhanced at adolescence compared to later in life, as this ability tends to be reduced with age (77). Further, several studies have demonstrated that TBI can itself promote bone formation, with enhanced fracture callus formation and heterotopic ossification both common following multitrauma involving TBI (48, 78, 79). As such, we speculate that our findings may also reflect the osteogenic effects of TBI, possibly due to injury-induced activation of bone morphogenic protein and/or neurotrophic signaling. While outside of the scope of the current study design, future experiments incorporating histological techniques are required to reveal the precise mechanisms underlying the observed changes to skull bone.

## Conclusion

Contrary to our hypothesis, our study found that a single mTBI during adolescence did not exacerbate the short-term neurobehavioral or neuropathological consequences of a subsequent mTBI in adulthood. Instead, a mild head impact during adolescence appeared to protect against glial reactivity in response to mTBI at adulthood, which may be partly but not completely attributed to group differences in the rate of skull fracture. Evidently, future studies are required to delineate the biological mechanisms underlying this difference.

Importantly, our findings highlight the process of active remodeling of the skull bones during adolescence and adulthood. Mechanical loading in the form of concussive or sub-concussive impacts to the head can influence skull bone structure and function, with possible consequences for how the skull and indeed brain itself responds to a subsequent injury. These findings may have important implications for young athletes exposed to mTBI, although it is clearly premature to consider clinical recommendations as a result of these findings. Nonetheless, our results do support the concept that sub-concussive impacts that do not result in any detectable neurobehavioral impairments may have consequences, either positive or negative, when repetitive in nature.

## Datasets are Available on Request

The raw data supporting the conclusions of this manuscript will be made available by the authors, without undue reservation, to any qualified researcher upon request.

## Ethics Statement

All procedures were performed in accordance with the guidelines of the Australian Code of Practice for the Care and Use of Animals for Scientific Purposes from the Australian National Health and Medical Research Council, and were approved by the Florey Institute of Neuroscience and Mental Health Animal Ethics Committee (#15-052-UM).

## Author Contributions

BS, SS, and SM conceptualized the study. TM, RB, LL, KW, MS, and BS performed the animal work and data collection. TM, RB, LL, and BS performed data analysis. BS, TO, SM, and SS provided interpretation of the study findings. TM, RB, and BS wrote the manuscript first draft, with critical review by SS, SM, and TO. All authors contributed to manuscript revision and approved the final version.

## Conflict of Interest Statement

The authors declare that the research was conducted in the absence of any commercial or financial relationships that could be construed as a potential conflict of interest.

## Footnotes

^1^https://www.random.org (Accessed: October 4, 2016).

^2^http://imagej.nih.gov/ij/ (Accessed: July, 2015).
