# Supplementary material for: Mild Traumatic Brain Injury in Adolescent Mice Alters Skull Bone Properties to Influence a Subsequent Brain Impact at Adulthood: A Pilot Study
Source: Front Neurol. 2018 May 25;9:372. doi: 10.3389/fneur.2018.00372 (PMC5980957; doi:10.3389/fneur.2018.00372)
Supplement: Supplementary file 1 [file data_sheet_1.docx]

**Supplementary Data 1**

**Optimization of weight-drop model for adolescent mice – pilot study**

The model has been best characterized in adult C57Bl/6 mice (10-12 weeks of age), with a drop height of 2 or 3 cm (Flierl et al. 2009). We chose 2 cm for the second impact at 10 weeks of age, as a mild severity of impact. The impactor has capacity for the rod to drop from 0.5 cm increments. To determine the most appropriate drop height for 5 week old (adolescent) mice, we first evaluated a 2 cm height, but it resulted in 100% acute mortality (n=4). A 1 cm drop height did not impact the skull at all, so was not a viable option. The 1.5 cm height drop resulted in visible impact to the exposed skull, with a period of apnea and delayed righting reflex (n=4) evident immediately post-impact compared to sham controls (n=2). We therefore chose this parameter for the rest of the study.

**Supplementary Data 2**

**Neurological Severity Score**

The Neurological Severity Score (NSS) is a broad assessment of general neurological function, including reflexes, balance and locomotion (Flierl et al. 2009). The test was performed by an investigator blinded to mTBI/sham, at 1 h post-surgery at P35 and P70. A score of 0 is given for completion of a task (e.g. balancing on a beam), or a score of 1 for failure to complete the task, for a total possible score of 10. No animal scored higher than a 2, and no differences in mean NSS were detected between groups (2-way ANOVA; n.s.). This indicates undetectable neurological impairments by the NSS scale as a result of mTBI impact in this model, at either age.

Supplementary Table 1: Neurological Severity Score at 1 h post-injury.

| **Group** | **At P35** | **At P70** |
| --- | --- | --- |
| Sham + Sham | 0.5 ± 0.3 | 0.2 ± 0.1 |
| P35 + Sham | 0.1 ± 0.1 | 0.4 ± 0.2 |
| Sham + P70 | 0.6 ± 0.2 | 0.1 ± 0.1 |
| P353 + P70 | 0.2 ± 0.1 | 0.2 ± 0.1 |

Mean NSS scores are presented ± standard error of the mean.

Flierl, M. A., et al. (2009). "Mouse closed head injury model induced by a weight-drop device." Nat Protoc **4**(9): 1328-1337.
